# Microvesicles Derived from Human Wharton's Jelly Mesenchymal Stem Cells Promote Human Renal Cancer Cell Growth and Aggressiveness through Induction of Hepatocyte Growth Factor

**DOI:** 10.1371/journal.pone.0096836

**Published:** 2014-05-05

**Authors:** Tao Du, Guanqun Ju, Shuai Wu, Zhongliang Cheng, Jun Cheng, Xiangyu Zou, Guangyuan Zhang, Shuai Miao, Guohua Liu, Yingjian Zhu

**Affiliations:** 1 Department of Urology, Shanghai First People's Hospital, School of Medicine, Shanghai Jiao Tong University, Shanghai, P.R. China; 2 Department of Urology, Henan Provincial People's Hospital, Zhengzhou, P.R. China; 3 Department of Urology, Qingdao Municipal Hospital, Qingdao, P.R. China; 4 Department of Urology, Nantong Tongzhou People's Hospital, Jiangsu Province, P.R. China; University of Torino, Italy

## Abstract

In our previous study, microvesicles (MVs) released from *human* Wharton's jelly mesenchymal stem cells (hWJ-MSCs) retard the growth of bladder cancer cells. We would like to know if MVs have a similar effect on *human* renal cell carcinoma (RCC). By use of cell culture and the BALB/c nu/nu *mice* xeno-graft model, the influence of MVs upon the growth and aggressiveness of RCC (786-0) was assessed. Cell counting kit-8 (CCK-8) assay, incidence of tumor, tumor size, Ki-67 or *TUNEL* staining was used to evaluate tumor cell growth *in vitro* or *in vivo*. Flow cytometry assay (*in vitro*) or examination of cyclin D1 expression (*in vivo*) was carried out to determine the alteration of cell cycle. The aggressiveness was analyzed by *Wound Healing Assay* (*in vitro*) or MMP-2 and MMP-9 expression (*in vivo*). AKT/p-AKT, ERK1/2/p-ERK1/2 or HGF/c-MET expression was detected by real-time PCR or western blot. Our data demonstrated that MVs promote the growth and aggressiveness of RCC both *in vitro* and *in vivo*. In addition, MVs facilitated the progression of cell cycle from G_0/1_ to S. HGF expression in RCC was greatly induced by MVs, associated with activation of AKT and ERK1/2 signaling pathways. RNase pre-treatment abrogated all effects of MVs. In summary, induction of HGF synthesis via RNA transferred by MVs activating AKT and ERK1/2 signaling is one of crucial contributors to the pro-tumor effect.

## Introduction

The ability of MSCs to home to sites of tumors has inspired investigation of these cells as potential anti-tumor tools [1], [2]. However, there has been debate about whether MSCs exert the anti-tumor effect until now. In quite a few of studies, MSCs do promote the progression of tumors. It has been documented that MSCs are not only involved in the formation of tumor microenvironment but interact with cancer cells to promote their growth and metastasis [3]–[5]. The exact mechanism of action remains unclear. Immunosuppressive action [6], [7], trans-differentiation toward the direction of mesenchymal cells [8], [9] as well as various bioactive factors secreted from MSCs [10] have been proposed to be potential effectors.

It has been recognized recently that MVs released from MSCs is one crucial effector of their actions. MVs have been described as a novel avenue for intercellular communication that may reprogram target cells by delivering functional mRNA and microRNA sequences [11], [12]. As MSCs are an abundant source of MVs, it is conceivable that they play a pivotal role in stem cell's biological effect [13]. Understanding the role of MVs in mediation of tumor cell behavior and function may aid in further disclosing the exact mechanisms that underlie MSCs pro- or anti-tumor effect.

In our previous study, MVs derived from hWJ-MSCs attenuated the growth of bladder tumor cell (T24) through down-regulation of AKT activation and up-regulation of cleaved Caspase-3 [14]. RCC is another common urologic tumor. We had a great interest in the influence of MVs on its growth and aggressiveness. *Human* renal cell carcinoma 786-0 cells were used in this study. Unlike T24, 786-0 cells express both HGF and c-MET, representing a cancer early progenitor cell marker [15]. Therefore, much attention is paid to the effect of MVs on the HGF/c-MET signaling pathway.

The *in vivo* and *in vitro* evidence indicates that MVs released by hWJ-MSCs favor the growth and aggressiveness of RCC. Further insight is given into the mechanisms underlying this effect. Data shows that induction of HGF expression in RCC via RNA information transferred by MVs is one of important contributors to activation of AKT and ERK1/2 signaling pathways which contribute to the pro-tumor effect of MVs. The medium conditioned by hWJ-MSCs has been demonstrated to induce native and foreign HGF synthesis in injured renal tubular cells [16]. In a recent study, we found that *human* HGF mRNA present in MVs derived from hWJ-MSCs is delivered into *rat* tubular cells subjected to hypoxia/re-oxygenation and is translated into the protein. We think that delivery of *human* HGF mRNA into *human* tumor cells may be one of mechanisms of action.

## Materials and Methods

### Ethics statement

In this study, all research involving human participants was approved by the institutional review board of the Chinese Academy of Medical Science and Medical School of Shanghai Jiao Tong University. Human individuals in this study gave written informed consent to participate in research and allow us to publish the case details. This study was carried out in strict accordance with the recommendations in the Guide for the Care and Use of Laboratory Animals of Shanghai Jiao Tong University. The protocol was approved by the Committee on the Ethics of Animal Experiments of Shanghai Jiao Tong University. All surgery was performed under sodium pentobarbital anesthesia, and all efforts were made to minimize suffering.

### Cell culture

This experiment was approved by the Research Ethics Committee at Shanghai Jiao Tong University Affiliated First People's Hospital. hWJ-MSCs were isolated and propagated as described before [17]. *Human* RCC line (786-0) (Shanghai Institutes for Biological Sciences, Shanghai, CHINA) was cultured in RPMI-1640 (Gibco) supplemented with 10% fetal bovine serum (FBS) (Gibco).

### Isolation and characterization of MVs

MVs released by hWJ-MSCs were isolated and characterized as previously described [14]. For the preparation of MVs, hWJ-MSCs were cultured in low-glucose DMEM deprived of FBS and supplemented with 0.5% bovine serum albumin (BSA) (Sigma-Aldrich, St. Louis, MO, USA) overnight. The supernatants were collected and centrifuged at 2000 g for 20 min to remove debris. The cell-free supernatants were ultra-centrifuged at 100000 g (Beckman Coulter Optima L-80K ultracentrifuge; Beckman Coulter, Fullerton, CA, USA) for 1h at 4°C. The supernatants were abandoned and the isolated MVs were suspended with M199 (Sigma-Aldrich) containing 25 mM HEPES (PH 7.4) and submitted to a second ultracentrifugation under the same conditions. MVs were re-suspended in serum-free M199. The protein content of MVs was quantified by *Bradford method* while *Limulus test* was employed to exclude endotoxin contaminations of MVs. RNA extracted from MVs by use of TRIZOL reagent was analyzed by spectrophotometer. Flow cytometric analyses of MVs showed the presence of CD9, CD29, CD44, CD63, CD73 and CD105, but not CD34 and CD45 (data not shown). The prepared MVs were stored at −80°C until further use.

### Transmission electron microscopy

The suspension was fixed with 2.5% glutaraldehyde in PBS for 2 h. After rinsing, MVs were ultra-centrifuged and suspended in 100 µl PBS. A 20 µl drop of MVs was loaded onto a formvar/carbon-coated grid, negatively stained with 3% aqueous phosphor-tungstic acid for 1min and observed by transmission electron microscopy (HITACHI, H-7650, Tokyo, JAPAN). The size of the isolated MVs ranged from 30 nm to 500 nm.

### MVs pre-treated with RNase

Part of isolated MVs were treated with 100 µg/mL RNase (Fermentas, Burlington, ON, CANADA) for 3 h at 37°C and the reaction was stopped by addition of RNase inhibitor (Fermentas). After ultracentrifugation at 100000 g for 1 h at 4°C, MVs were suspended by M199 and then stored at −80°C until use (RNase-MVs). Spectrophotometer analysis revealed that most of RNA extracted from MVs by use of TRIZOL reagent (Invitrogen, Carlsbad, CA, USA) was degraded by RNase treatment (MVs: 1.8±0.3 µg RNA/mg protein; RNase-MVs: less than 0.15 µg RNA/mg protein).

### Animal model

Eighteen male BALB/c nu/nu mice of 4–6 wk years old (Laboratory Animal Center of Shanghai, Academy of Science, Shanghai, CHINA) were randomly divided into 3 groups (n = 6 for each group). All mice received subcutaneous injection of 1×10^7^ 786-0 cells with the addition of MVs (200 µg/ml protein)(an amount of MVs released by approximately 1×10^6^ hWJ-MSCs overnight), RNase-MVs or M199 (control). Animals were monitored every 3 days. The time-point of tumor occurrence was recorded. Tumor growth was evaluated by tumor volume, which was calculated by the modified ellipsoidal formula: V = 1/2 (length × width) [18]. The length and width of tumor mass was measured by caliper.

### CCK-8 assay

CCK-8 (Beyotime institute of biotechnology, Jiangsu, CHINA) was used to determine *in vitro* growth of tumor cells. 786-0 cells were seeded in 96-well plates (2000cells/well) and incubated in a humidified atmosphere containing 5% CO_2_ at 37°C. After 24 h of incubation, cells were washed with PBS, and then co-cultured with MVs (200 µg/ml protein), RNase-MVs in M199 or M199 (control) for 24 or 48 h. Cells were washed twice with PBS and incubated in 100 µl RPMI-1640 (Gibco) containing 10 µl CCK-8 reagent for another 3 h. The absorbance of each well at 450 nm was spectrophoto-metrically measured using a micro-plate ELISA reader (Bio-Tek Instruments, Winooski, VT, USA). Culture medium (RPMI-1640) without cells was used as blank control.

### Analysis of cell cycle and Wound Healing Assay

786-0 cells (5×10^4^) were incubated with MVs (200 µg/ml) or RNase-MVs in M199 or M199 (control) for 48 h. After treatment with trypsin, cells were collected and washed twice with PBS, then fixed in 70% ice-cold ethanol for 24 h. The fixed cells were stained with 50 µg/ml propidium iodide (PI) (Sigma) in PBS containing 0.1% Triton X-100 (Sigma) and 50 µg/ml RNase (Sigma), and then analyzed of G_0_/G_1_, G_2_/M and S phase rate by a FACS Calibur flow cytometer (BECTON DICKINSON FACS Calibur, Franklin Lake, NJ, USA).

*Wound Healing Assay* was also performed to assess cell migration. 786-0 cells (5×10^4^) were seeded in 6-well plates in fresh RPMI-1640 media (Gibco) for 12 h of initial culture, and then continued to culture for another 12 and 24 h with the addition of MVs or RNase-MVs in M199 or M199 (control). The basic steps involve creating a “wound” in a cell monolayer, capturing the images at the beginning and at the end during cell migration to close the wound. The wound was created by manually scraping the cell monolayer with a p200 pipet tip. The area of the wounded region lacking cells was recorded for evaluation.

### Real time PCR

Total RNA was isolated from cell or tumor samples using the Trizol reagent (Invitrogen, Carlsbad, CA, USA) and then was reversely transcribed with the Moloney murine leukemia virus (M-MLV) reverse transcriptase Kit (Promega, Madison, WI, USA) and Oligo dT primers (Invitrogen, Carlsbad, CA, USA) for 60 min at 42°C. Real-time PCR was performed by TaqMan gene expression assays (Applied Bio-Systems, Foster City, CA, USA) for detection of gene expression of HGF, MMP-2, MMP-9 or β-actin using the following primers:

*HGF*: 5′CTCTGGTTCCCCTTCAATAG, 3′GATAGCCCCATTTCTGGATGTC;

*MMP-2*:5′CCCATTTTGATGACGATGA, 3′CAAGTTACCGTTCCTCATGTT;

*MMP-9*:5′CGAACTTTGACAGCGACAAGA, 3′GATACCAGGAGCGGGACTT;

*β-actin*: 5′AAGGTGACAGCAGTCGGTT, 3′GGAGAGGGTTCAGGTGTGT;

The quantification of the target gene was normalized by β-actin, an internal control gene. The Ct for the target gene and β-actin was determined for each sample. The experimental samples were expressed as an n-fold difference relative to the control (M199-treated cell or tumor samples). The relative expression of mRNA expression was calculated by 2^−ΔΔCT^. Triplicates of each sample were performed.

### Western blot analysis

Protein extracts (30 µg per lane) were electrophoresed and then transferred to polyvinylidene fluoride membrane. Immuno-blotting was performed by incubating each membrane with an anti-HGF (dilution: 1∶1000; Abcam, Cambridge, UK), c-MET (dilution: 1∶250; Abcam, Cambridge, UK), AKT (dilution: 1∶1000; Abcam, Cambridge, UK), p-AKT (dilution: 1∶1000; Abcam, Cambridge, UK), ERK1/2 (dilution: 1∶1000; Abcam, Cambridge, UK), p-ERK1/2 (dilution: 1∶1000; Abcam, Cambridge, UK), MMP-2 (dilution: 1∶1000; Abcam, Cambridge, UK), MMP-9 (dilution: 1∶500; Abcam, Cambridge, UK), cyclin D1 (dilution: 1∶250; Abcam, Cambridge, UK) or β-actin overnight at 4°C. After being washed in PBS, each membrane was incubated for 1 h with a secondary antibody conjugated by peroxidase at room temperature. The band was developed by use of enhanced chemi-luminescence (Amersham Pharmacia Biotech, Piscataway, NJ, USA). The density of each band was determined. The results were repeated twice to confirm the reproducibility.

### Immunohistochemistry

Briefly, the slides were incubated with antibody to human HGF (dilution: 1∶100; Abcam, Cambridge, UK) or Ki-67 (dilution: 1∶200; Abcam, Cambridge, UK) followed by a HPR-conjugated secondary antibody by use of DAB as substrate. Negative control was performed by replacing primary antibodies with a non-immune immunoglobulin of the same iso-type. Nuclei were counterstained with Harris hematoxylin. Cell apoptosis was assessed by a terminal transferase-mediated dUTP nick-end labeling (TUNEL) assay using an *In Situ Cell Death Detection Kit* (Roche, Mannheim, Germany). All sections were reviewed by a doctor blinded to this experiment.

### Supplementary experiments

Thirty male BALB/c nu/nu mice of 4–6 wk years old were randomly subcutaneously inoculated with 1×10^7^ 786-0 cells with the addition of conditioned medium (CM) from hWJ-MSCs, MVs isolated from CM or control medium (M199 or low-glucose DMEM). Animals were monitored every 3 days. The time-point of tumor occurrence and tumor size was recorded. *In vitro*, 786-0 cells were seeded in 96-well plates (2000 cells/well) and incubated in a humidified atmosphere containing 5% CO_2_ at 37°C. After 24 h of incubation, cells were washed with PBS, and then co-cultured with CM, MVs isolated from CM or control medium for 24 or 48 h. CCK-8 Assay was performed to determine cell growth.

In another supplementary experiment, 786-0 cells (5×10^4^) were seeded in 6-well plates in fresh RPMI-1640 media (Gibco) for 12 h of initial culture, and were then incubated with MVs (200 µg/ml protein), MVs with addition of PF2341066 (Pfizer, 6 µM), PF2341066 or control medium for 48 h. Total proteins in cells were isolated for western blot analysis of p-AKT and p-ERK1/2. The effects of various treatments on cell growth were also assessed by CCK-8 Assay.

### Statistical analysis

Data are reported as the means ± standard deviation (SD). Statistical analyses were performed using *SPSS v19.0.* Statistical difference between data means was determined by 2-tailed *Student's t test* and *analysis of variance* (ANOVA) as appropriate. A value of *P<0.05* was considered to be statistically significant.

## Results

### MVs facilitate the growth and migration of 786-0 cells in vitro

**Figure 1:**
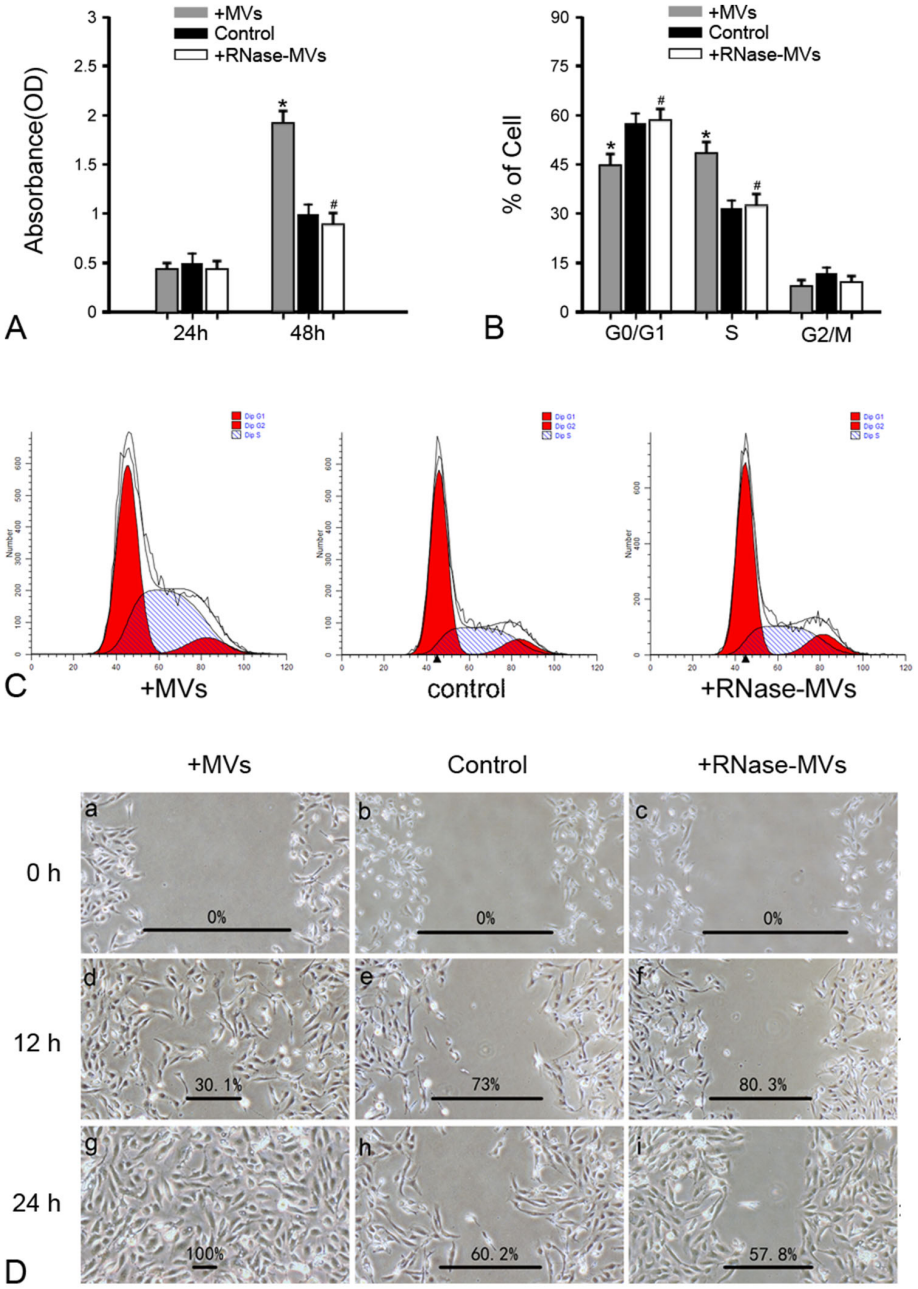
MVs promote the growth and migration of 786-0 cells in vitro. **(A) CCK-8 Assay.** After 48-0 cells, MVs greatly promoted cell growth whereas pretreatment with RNase abrogated this effect. The cells incubated with M199 were regarded as control. *P<0.01, MVs vs. control; ^#^ *P*<0.05, RNase-MVs vs. MVs; **(B) (C) Cell cycle analysis.** 786-0 cells treated with MVs primarily accumulated in S phase as compared to G_0_/G_1_ phase in RNase-MVs or control group. *P<0.05, MVs vs. control; ^#^ *P*<0.05, RNase-MVs vs. MVs; **(D)** ***Wound Healing Assay*** **.** The migration ability of 786-0 cells was significantly amplified by MVs.

The CCK-8 assay revealed that MVs greatly promote the proliferation of 786-0 cells after 48 h of incubation whereas RNase pre-treatment abrogated this effect (Fig. 1A). Analysis of cell cycle also showed that 786-0 cells treated with MVs predominantly accumulated in S phase as compared to G_0_/G_1_ phase in RNase-MVs or control group (Fig. 1B and 1C). Based on these results, we believe that MVs facilitate the progression of cell cycle, thereby accelerating the proliferation of RCC. In addition, *Wound Healing Assay* was employed to assess the ability of cell migration. Data indicated that MVs prominently enhance cell migration (Fig. 1D).

### MVs accelerate the development and progression of inoculated tumors

**Figure 2:**
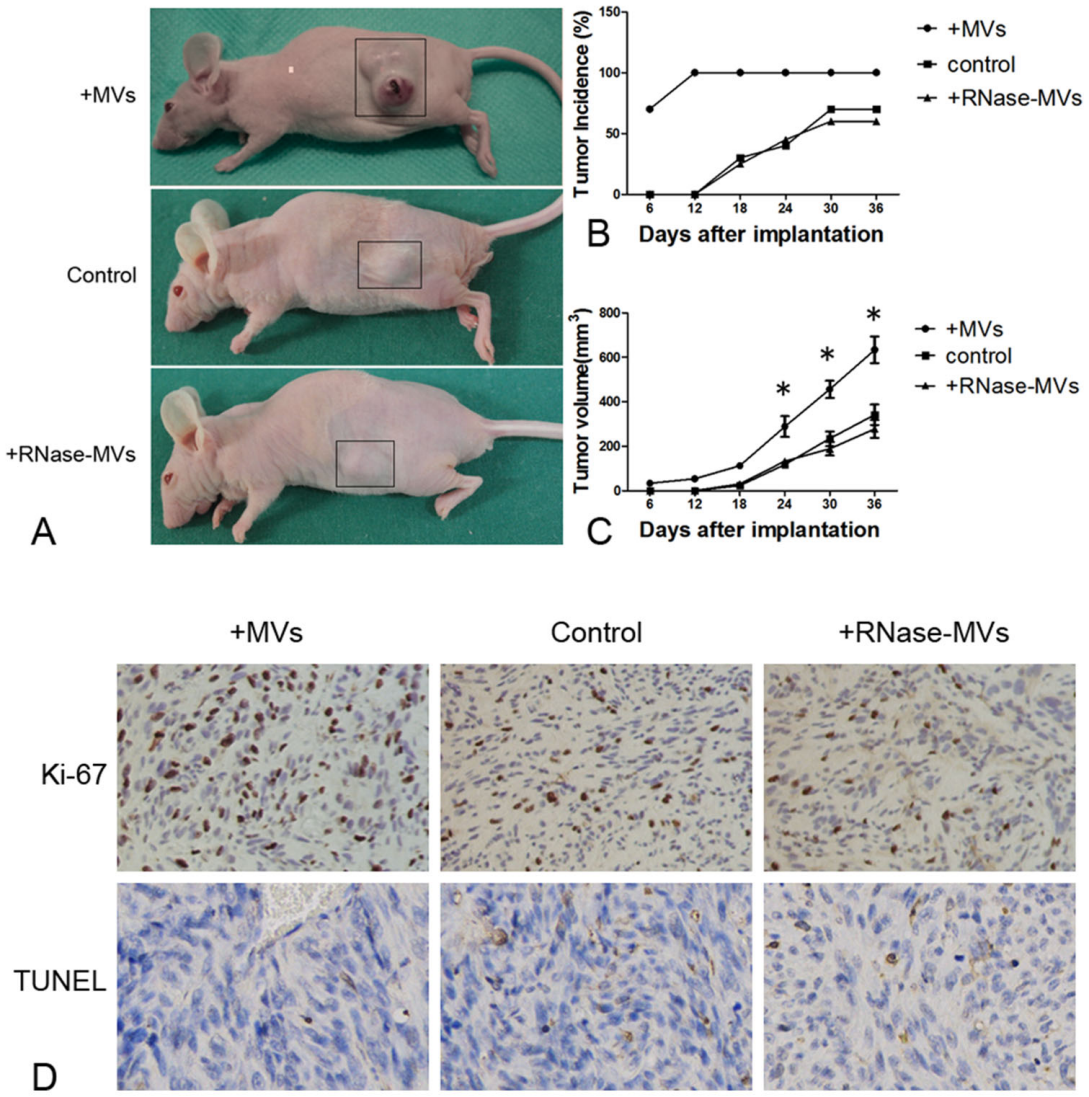
MVs facilitate the growth of inoculated tumors. **(A) Nude mice inoculated with 786-0 cells.** 786-0 cells mixed with MVs or RNase-MVs in M199 or M199 (control) were inoculated subcutaneously in the nude mice sacrificed at 36d following inoculation; **(B) Tumor incidence.** There was a higher tumor incidence in nude mice treated with MVs as compared to RNase-MVs or control; **(C) Tumor volume.** In contrast to RNase-MVs or control, MVs led to the development of a bigger tumor in nude mice. *P<0.05, MVs vs. RNase-MVs or control; **(D) Ki-67 and TUNEL staining.** In the presence of MVs, there were more Ki-67-positive staining cells and fewer cells staining positive for TUNEL on tumor sections.

**Figure 3:**
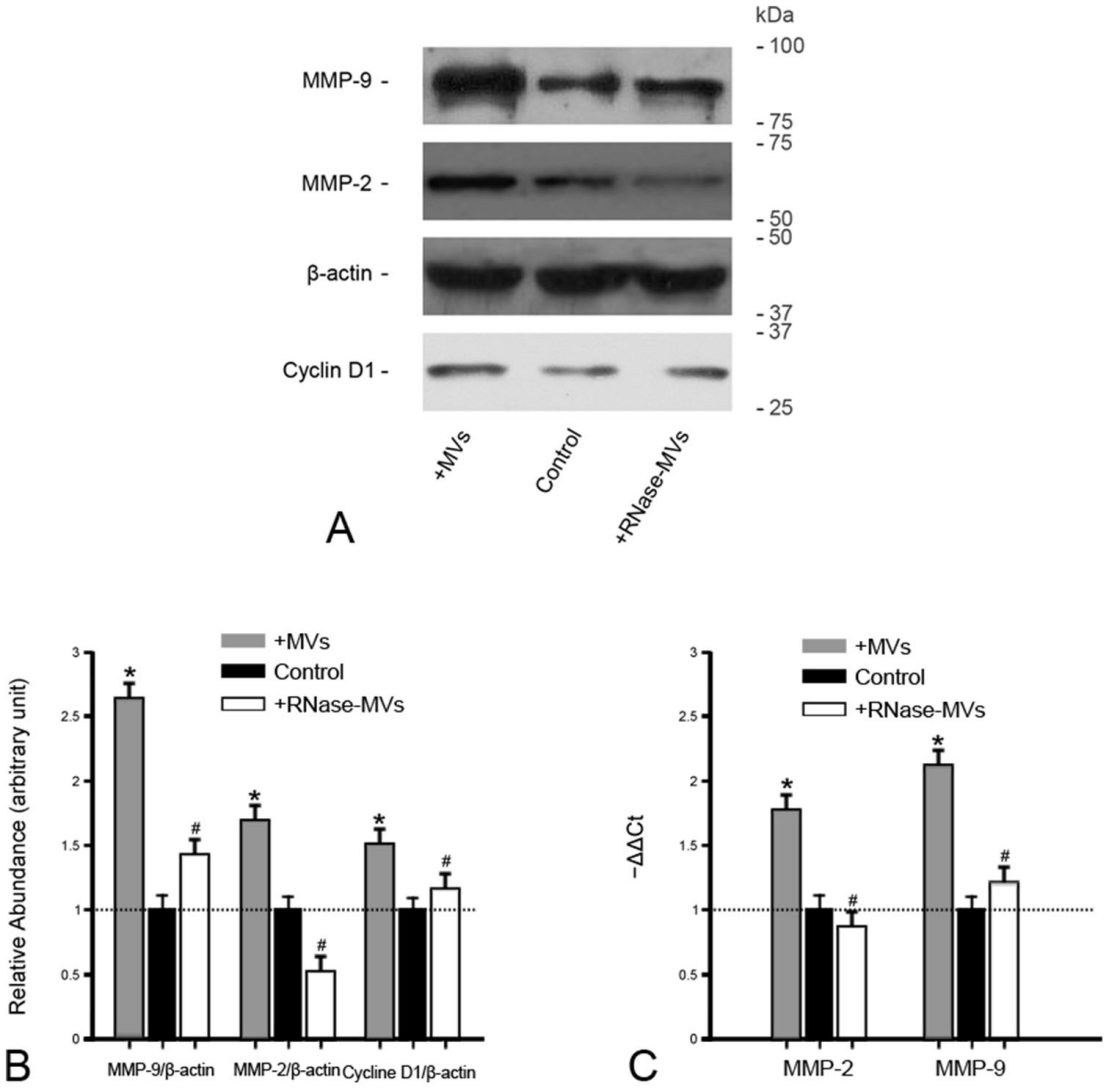
MVs enhance the expression of cyclin D1, MMP-2 and MMP-9 in tumor tissues. **(A)(B) Gel photograph and densito-metric analysis of cyclin D1, MMP-2 and MMP-9 protein expression.** MVs led to a significant up-regulation of cyclin D1, MMP-2 and MMP-9 protein expression in tumor tissues. RNase pretreatment destructed these effects. The density of each band was determined. Values in the graph are expressed as densitometry ratios of cylin D1/β-actin, MMP-2/β-actin or MMP-9/β-actin as folds over control (dotted line). Data were obtained from 3 animals for each experiment. *P<0.05, MVs vs. control; ^#^ *P*<0.05, RNase-MVs vs. MVs; **(C) Gene expression of MMP-2 and MMP-9.** The gene expression of MMP-2 or MMP-9 was markedly up-regulated by MV**s.** The Ct (threshold cycle) for each gene was determined for each sample. The quantification of MMP-2 or MMP-9 was normalized by β-actin. The gene expression in control group was regarded as the calibrator (dotted line).The relative expression of the target gene was calculated by 2^−ΔΔCt^. *P<0.05, MVs vs. control; ^#^ *P*<0.05, RNase-MVs vs. MVs.

In the presence of MVs, tumor nodules were formed in some of mice as early as 6d after inoculation with 786-0 cells (Fig. 2B). By 18d, tumor incidence reached 100% (Fig. 2B). By contrast, there was no formation of tumor nodules in mice treated with control medium until 12d after inoculation (Fig. 2B). The final tumor incidence was only 75% (Fig. 2B). In addition, tumor growth under the induction of MVs was faster than that induced by RNase-MVs or vehicle as indicated by measurement of tumor size (Fig. 2A and 2C). Moreover, more Ki-67-positive and fewer TUNEL-positive tumor cells were detected on tumor sections from mice receiving treatment with MVs (Fig. 2D). Of interest, cyclin D1 protein expression in tumor tissues was markedly up-regulated by MVs (Fig. 3A and 3B). Cyclin D1, as a critical mediator, is involved in transition of cell cycle from G_1_ to S phase. Therefore, the increase in cyclin D1 expression facilitates cell cycle progression. The expression of MMP-2 and MMP-9 is used to assess the ability of tumor aggressiveness. By means of RT-PCR and western blot, we found that MVs lead to a significant up-regulation of their gene and protein expression (Fig. 3A–3C). All these evidence shows that MVs promote tumor development and progression, in agreement with the results of *in vitro* experiment.

### Activation of AKT and ERK1/2 signaling in inoculated tumors is elicited by MVs

**Figure 4:**
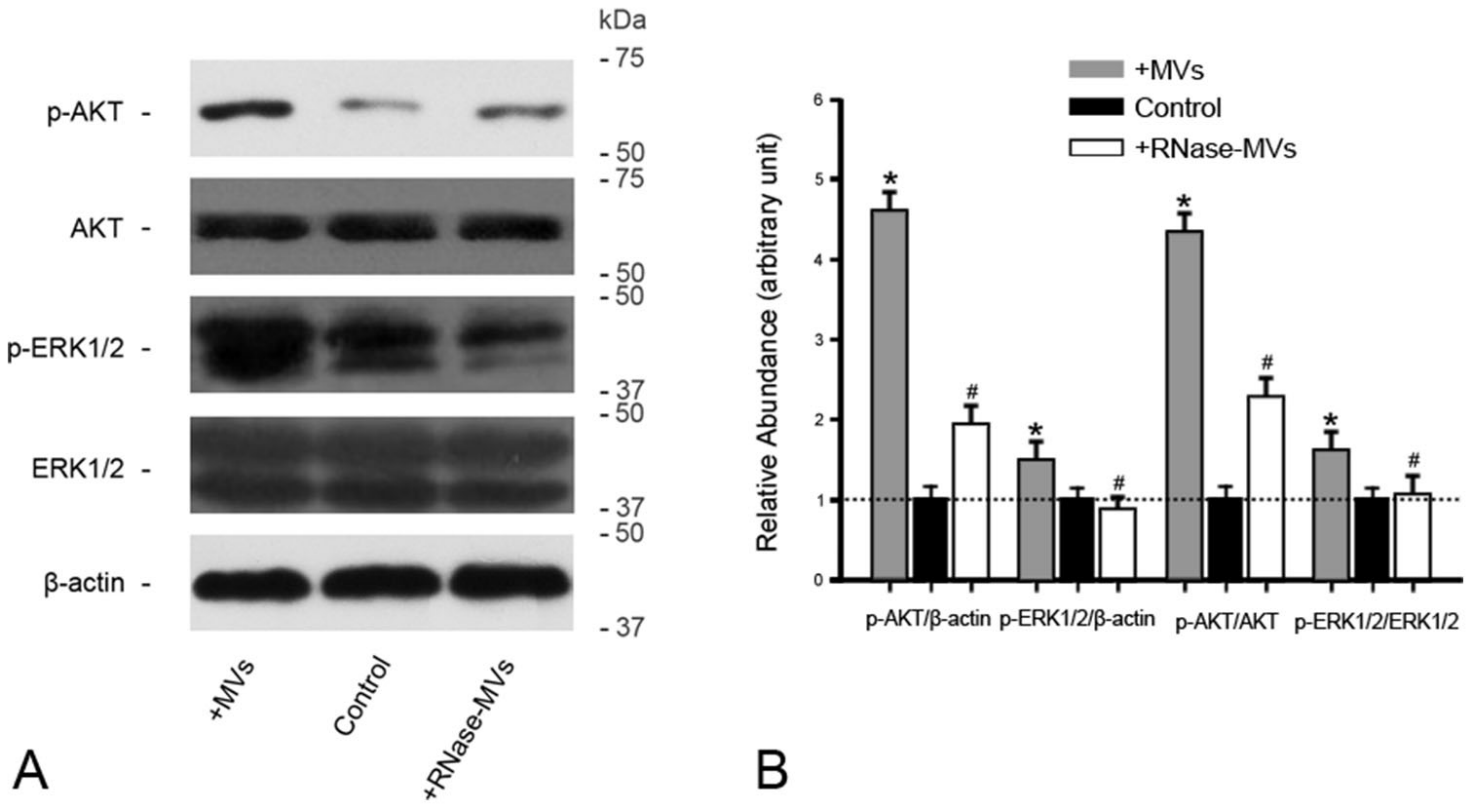
MVs induce the activation of AKT and ERK1/2 signaling in tumor tissues. **(A)(B) Gel photograph and densito-metric analysis of of AKT, p-AKT, ERK1/2 and p-EREK1/2 protein expression in inoculated tumors.** MVs led to a significant phosphorylation of tumor AKT and ERK1/2 proteins. The density of each band was determined. Values in the graph are expressed as densitometry ratios of p-AKT/β-actin, p-ERK1/2/β-actin, p-AKT/AKT or p-ERK1/2/ERK1/2 as folds over control (dotted line). *P<0.05, MVs vs. control; ^#^ *P*<0.05, RNase-MVs vs. MVs.

In view of the close association of AKT and ERK1/2 signaling with survival, growth and migration of RCC [19], [20], we also determined the alteration of these two signaling elicited by MVs in the *in vivo* experimental setting. Western blot analysis revealed that, MVs led to a robust increase in phosphorylation level of AKT in inoculated tumors in contrast to RNase-MVs or control (Fig. 4A and 4B). MVs also caused a comparatively weaker but significant elevation of p-ERK1/2 level (Fig. 4A and 4B).

### MVs induce HGF expression in RCC either in vitro or in vivo

It has been documented that HGF/c-MET are involved in cancer cell proliferation and invasion [21], [22]. In addition, over- or mis-expression of HGF and c-MET often correlates with poor prognosis and metastasis of human RCC [23]–[26]. HGF/c-MET signaling can also lead to activation of both AKT and ERK1/2 signaling. Hence, we made further investigation into the impact of MVs upon HGF/c-MET expression in RCC.

**Figure 5:**
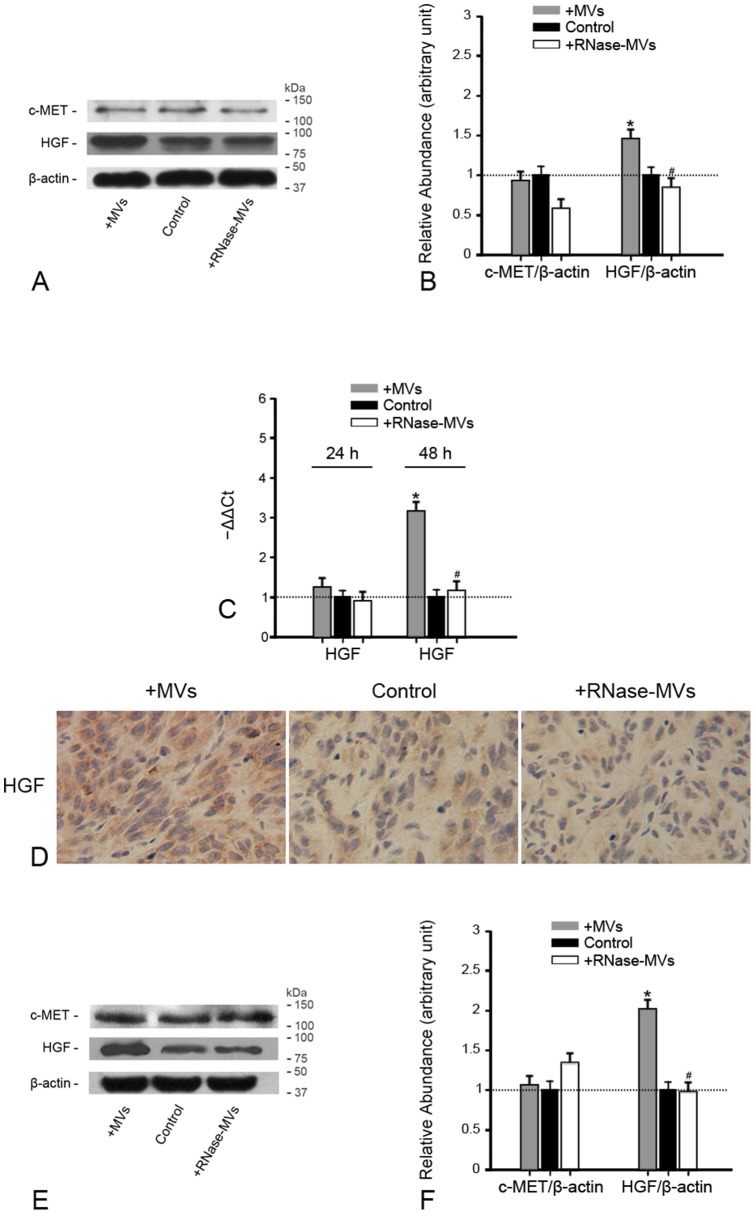
MVs induce HGF expression in RCC either *in vivo* or *in vitro*. **(A)(B) Gel photograph and densitometric analysis of HGF and c-MET protein expression in 786-0 cells. At 48 h following the addition of MVs, HGF protein expression in 786-0 cells was prominently up-regulated while c-MET expression underwent no significant change. Pre-treatment with RNase destructed the effect of MVs.** The density of each band was determined. Values in the graph are expressed as densitometry ratios of HGF/β-actin or c-MET/β-actin as folds over control (dotted line). *P<0.05, MVs vs. control; ^#^ *P*<0.05, RNase-MVs vs. MVs; **(C) HGF gene expression in 786-0 cells. After 48 h of incubation with 786-0 cells, MVs substantially induced the up-regulation of HGF gene expression, frustrated by RNase treatment.** The Ct (threshold cycle) for each gene was determined for each sample. The quantification of HGF was normalized by β-actin. HGF expression in control group was regarded as the calibrator (dotted line). The relative expression of the target gene was calculated by 2^−ΔΔCt^. *P<0.05, MVs vs. control; ^#^ *P*<0.05, RNase-MVs vs. MVs; **(D) HGF staining on tumor sections. There was a significant intensification of HGF staining on tumor sections in the setting of MVs as compared to RNase-MVs or control; (E)(F) Gel photograph and densitometric analysis of HGF and c-MET protein expression in tumor tissues.** MVs greatly induced HGF protein expression in tumor tissues. As *in vitro*, pretreatment with RNase abrogated the inductive effect. *P<0.05, MVs vs. control; ^#^ *P*<0.05, RNase-MVs vs. MVs.

After 48 h of incubation with MVs, HGF protein expression in 786-0 cells was substantially up-regulated as demonstrated by western blot analysis whereas c-MET expression did not undergo such a change (Fig. 5A and 5B). A similar result was achieved by examination of HGF mRNA expression (Fig. 5C). The inductive effect of MVs on HGF is also verified in *in vivo* experimental setting. In the presence of MVs, there was a marked intensification of HGF staining on tumor sections (Fig. 5D). Western blot analysis also revealed that the addition of MVs led to a marked increase of HGF protein expression in tumor tissues (Fig. 5E–5F). As *in vitro*, c-MET protein expression in inoculated tumors did not be affected by MVs (Fig. 5E–5F). Pretreatment with RNase expectedly frustrated the alterations in HGF expression either *in vivo* or *in vitro*, implying the crucial role of RNA information delivered by MVs in HGF induction. Since HGF induction is in parallel with activation of AKT and ERK1/2, MVs may trigger the activation of AKT and ERK 1/2 via HGF induction, at least in part.

### *In vitro*, c-Met inhibitor abrogates the activation of AKT and ERK1/2 signaling in 786-0 cells and cell growth induced by MVs

**Figure 6:**
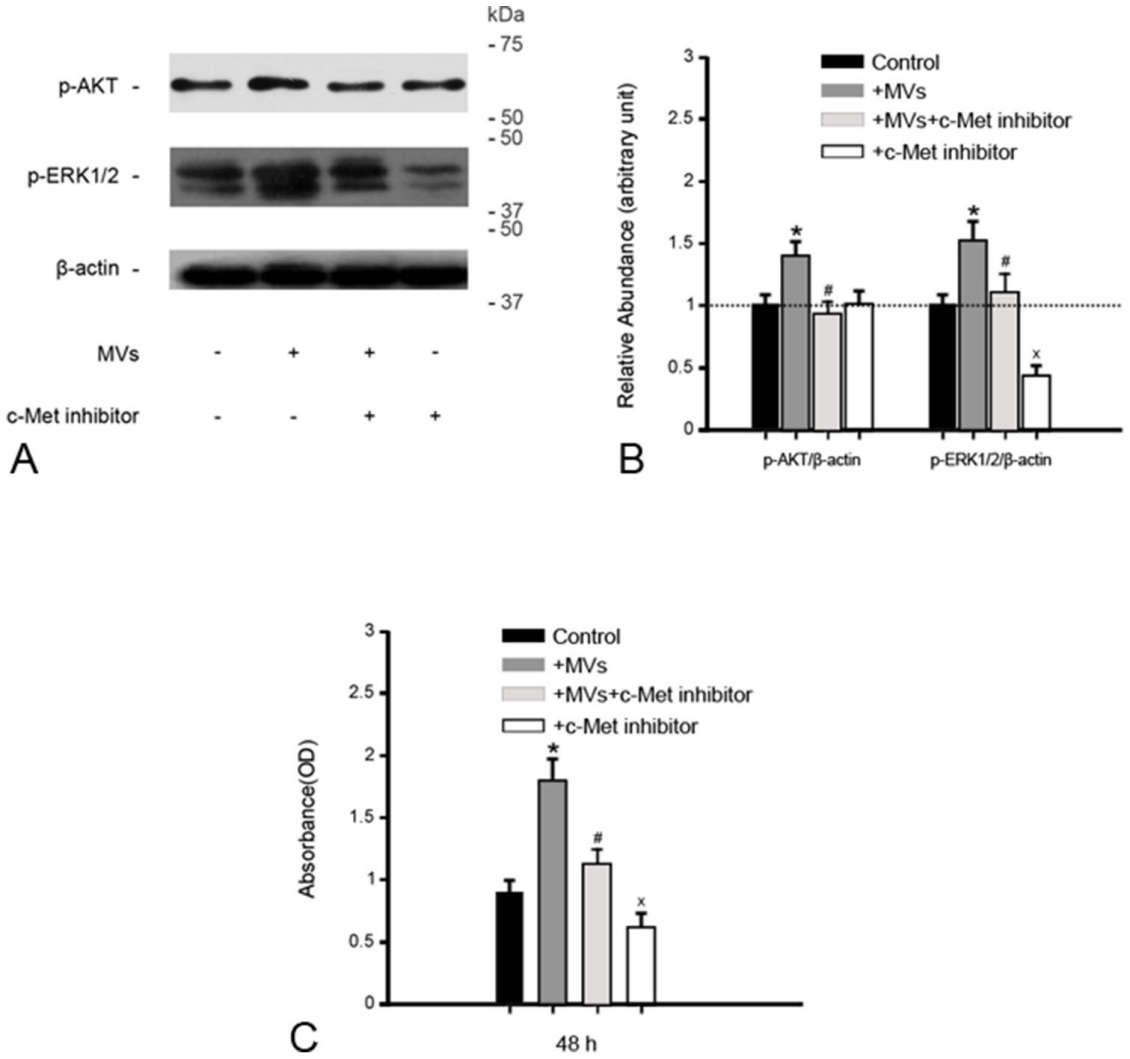
c-Met inhibitor abrogates the activation of AKT and ERK1/2 signaling in 786-0 cells and cell growth induced by MVs. **(A)(B) Gel photograph and densitometric analysis of p-AKT and p-ERK1/2 protein expression in 786-0 cells.** MVs led to marked phosphorylation of AKT and ERK1/2 proteins after 48 h incubation with 786-0 cells, abolished by c-Met inhibitor. The density of each band was determined. Values in the graph are expressed as densitometry ratios of p-AKT/β-actin, p-ERK1/2/β-actin as folds over control (dotted line). *P<0.05, MVs vs. control; ^#^ *P*<0.05, MVs+c-Met inhibitor vs. MVs; ^×^ *P*<0.05, c-Met inhibitor vs. control; **(C) CCK-8 Assay.** After 48 h incubation with 786-0 cells, the addition of c-Met inhibitor greatly retarded cell growth induced by MVs. *P<0.01, MVs vs. control; ^#^ *P*<0.05, MVs+c-Met inhibitor vs. MVs; ^×^ *P*<0.05, c-Met inhibitor vs. control.

As *in vivo*, MVs led to a marked activation of AKT and ERK1/2 signaling in 786-0 cells after 48 h of incubation (Fig. 6A and 6B). For verification of the association of HGF induction with activation of AKT and ERK1/2, c-Met inhibitor was added to block the HGF/c-Met signaling pathway. Of interest, with the addition of c-Met inhibitor, activation of AKT and ERK1/2 signaling induced by MVs was substantially frustrated (Fig. 6A and 6B), indicating that HGF induction is one of critical contributors to activation of AKT and ERK1/2 signaling. Furthermore, c-Met inhibitor significantly retarded tumor cell growth stimulated by MVs as shown by CCK-8 (Fig. 6C).

### The medium conditioned by hWJ-MSCs have a similar pro-tumor effect as MVs both *in vivo* and *in vitro*

**Figure 7:**
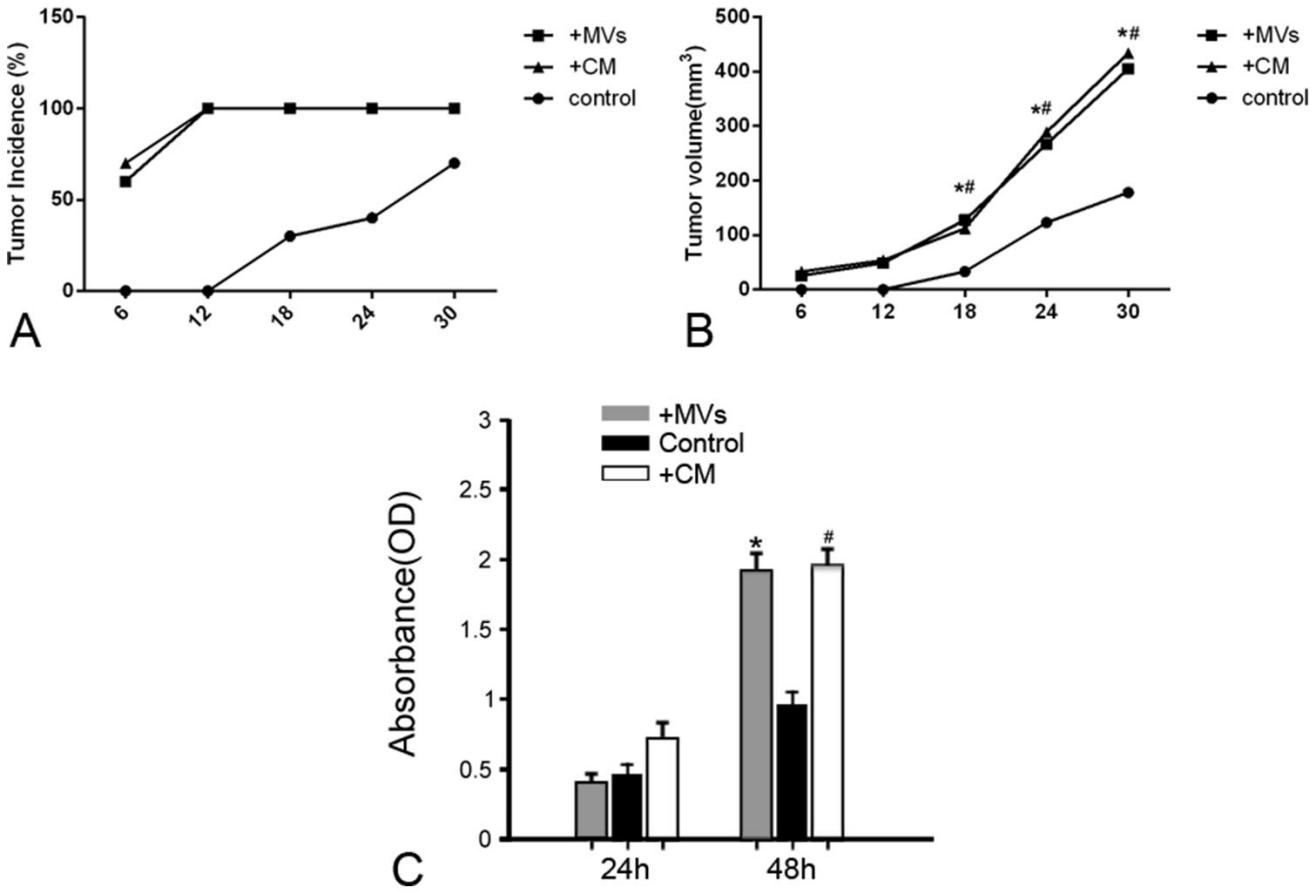
The medium conditioned by hWJ-MSCs have a similar pro-tumor effect as MVs both *in vivo* and *in vitro*. **(A) Tumor incidence.** There was a higher tumor incidence in nude mice treated with CM or MVs as compared with control; **(B) Tumor volume.** In contrast to control, CM or MVs led to the development of a bigger tumor in nude mice. *P<0.05, MVs vs. control; ^#^ *P*<0.05, CM vs. control; (C) **CCK-8 Assay.** After 48 h incubation with 786-0 cells, CM or MVs greatly promoted tumor cell growth. *P<0.01, MVs vs. control; ^#^ *P*<0.01, CM vs. control.

In contrast to control, the conditioned medium (CM) of hWJ-MSCs led to earlier tumor occurrence and bigger tumor size (Fig. 7A and 7B). CCK-8 Assay also revealed that CM greatly accelerated the growth of 786-0 cells as *in vivo* (Fig. 7C). On basis of these results, we ensure that MVs are one of crucial effectors of hWJ-MSCs. To explore the mechanisms of action of MVs may aid in disclosing how hWJ-MSCs exert the pro-tumor effect on RCC.

## Discussion

The function of MVs as new mediators on cell-to-cell communication has been demonstrated in several studies [27]–[29]. A horizontal transfer of functional mRNAs and mi-RNAs via MVs is involved in the genetic exchange between cells [30]–[32]. The genetic information transferred by MVs is the main effector of functional and pheno-typical changes occurring in recipient cells [27], [30], [33]. In animal models of kidney injury, MVs mimic the beneficial effect of MSCs. Accordingly MVs released by MSCs may be one critical contributor to their pro- or anti-tumor properties. To explore the molecular alteration in tumor cells elicited by MVs does favor to the disclosure of the essence of MSCs' action.

Some researchers have reported that MVs derived from human MSCs exert anti-tumor effect in *in vivo* and *in vitro* experimental setting [34], [35]. In our previous study, MVs released by hWJ-MSCs also prevented bladder tumor cell growth. RCC is another common urologic tumor. We were very curious about the impact of MVs on this tumor. The in vitro and in vivo experiments were carried out to resolve this issue.

We successfully isolated MVs from hWJ-MSCs' supernatant and characterized them. MVs were composed of hetero-geneous lipid bi-layer vesicles ranging from 30–500 nm and expressed CD9, CD29, CD44, CD63, CD73 and CD105. MVs were rich in RNA (1.8±0.3 µg RNA/mg protein) and RNase pretreatment nearly completely (>90%) destructed the RNA content.

By use of various methods, we made clear the influence of MVs upon the growth of RCC. *In vitro*, MVs promote the growth of 786-0 as demonstrated by CCK-8 assay. In the presence of MVs, formation of RCC tumor was more rapid while tumor size was bigger. In addition, there were more proliferating tumor cells and fewer cells in apoptosis on histologic examination. The alteration of cell cycle triggered by MVs was also determined. MVs led to accumulation of 786-0 cells primarily in the S phase rather than G0/G1 phase. *In vivo*, cyclin D1 protein expression in tumor tissues was also greatly up-regulated. Cyclin D1, as a critical mediator, is involved in transition of cell cycle from G1 to S phase [36]. It is conceivable that MVs promote RCC growth through facilitation of tumor cell cycle progression. A similar phenomenon is also observed in some animal models of acute renal or liver injury. Administration of MVs released from stem cells induces a cell cycle re-entry of injured epithelial cells via activation of regenerative programs [37]–[40].

On the other hand, we found that MVs favor RCC invasion. *In vitro*, *Wound Healing Assay* distinctly identified the amplification of cell invasive ability under the induction of MVs. In addition, expression of MMP-2 and MMP-9 in tumor tissues was markedly enhanced by MVs. The expression level of MMPs has been confirmed to a positive correlation with tumor progression in various types of cancer [41]. *In vitro*, the induction of MMPs also participates in the 3-D spreading and invasion of cancer cells [42]. Among MMPs, MMP-2 and MMP-9 are regarded as the critical indicators for tumor cell invasion and metastasis [43], [44]. Hence, the increased expression facilitates tumor aggressiveness.

Given the close association of AKT and ERK1/2 signaling with tumor cell survival, growth and invasion [19], [20], we further examined their change caused by MVs. Through western blot analysis, we found that MVs substantially promote the activation of AKT and ERK1/2 signaling in RCC tumor. The activation of AKT and ERK1/2 signaling induced by MVs has been reported by some researchers. 1) Embryonic stem cell-derived MVs (ES-MVs) can reprogram hematopoietic progenitors and induce phosphorylation of MAPK (ERK1/2) and AKT in murine Sca-1^+^cells [45]; 2) Endothelial progenitor cell–derived MVs also activate an angio-genic program in endothelial cells by a horizontal transfer of mRNA associated with the PI3K/AKT signaling pathway [31].

We are eager to know how MVs exert all these effects: 1) activation of AKT and ERK1/2 signaling; 2) facilitation of cell cycle progression from G0/G1 to S phase; 3) enhancement of MMP-2 and MMP-9 expression. Through review of some related articles, we assumed that activation of HGF/c-MET signaling may be one of prime pathways. 1) HGF binding to c-MET leads to phosphorylation of two tyrosine residues at the C-terminus of MET, which in turn leads to the activation of the PI3K/AKT and Ras/MAPK (ERK1/2) effector pathways, thereby promoting RCC growth and metastasis [19]; 2) Active ERK1/2 translocate to the nucleus, where they phosphorylate and stabilize several transcription factors that are involved in the early phases of the G1→S cell cycle transition; In addition, activation of AKT inactivates glycogen synthase kinase 3-β (GSK3-β), which antagonizes the expression of cyclin D1; 3) HGF has an important role in the regulation of the functions of MMPs by enhancing the transcriptional levels of a large number of MMPs as well as those of several proteases that convert the precursor forms of MMPs into active enzymes [46], [47].

Inspiringly, MVs derived from hWJ-MSCs do induce the up-regulation of HGF gene and protein expression in RCC. *In vitro*, after 48 h incubation with MVs, HGF gene and protein expression in 786-0 cells was prominently increased. In BALB/c-nu/nu mice receiving the inoculation of 786-0 cells, HGF protein expression in tumor tissues was markedly enhanced by MVs, which was further corroborated by a marked intensification of HGF staining in tumor cells. As pre-treatment of RNase completely frustrated the induction of HGF, it is assumed that RNA delivered by MVs is one crucial mediator. In our recent study, *human* HGF mRNA present in MVs was delivered into *rat* tubular cells subjected to hypoxia/re-oxygenation and was translated into the corresponding protein. It has been established that the effective horizontal transfer of mRNA or microRNA via MVs can reprogram the target cells and thus alter their phenotype and functions [35], [45]. In our opinion, delivery of HGF mRNA into tumor cells may be one of inductive mechanisms. Further insight should be given into the exact mechanisms implicated in HGF induction. In this study, c-Met expression was not altered by MVs. Although MVs have the potential to transmit receptors to the target cells [48], MVs from hWJ-MSCs do not deliver c-Met receptor to RCC, presumably due to absence of c-Met expression in cells of origin.

For verification of the close relationship of HGF induction with activation of AKT and ERK1/2 signaling, the supplementary experiment was carried out. We found that MVs lead to activation of AKT and ERK1/2 as *in vivo*. c-Met inhibitor robustly frustrated the effect of MVs, indicating that HGF induction is one of important contributors to activation of AKT and ERK1/2 signaling. It has been documented that MSC-derived exosomes promote *in vivo* growth of *human* gastric carcinoma and *human* colon cancer via activation of ERK1/2 pathway [49]. It seems that there is a common pathway for ERK1/2 activation. Further study is required to corroborate this speculation.

In this study, we provide some substantial evidence that MVs derived from hWJ-MSCs can promote RCC growth and invasiveness. The molecular mechanism involved in the pro-tumor effect is also disclosed. The induction of HGF via RNA transfer activating AKT and ERK1/2 signaling is one of critical mechanisms. This inductive effect of MVs is very attractive. We envision that it might be exploited in regeneration medicine. Through induction of HGF synthesis in damaged tissue cells, MVs might ameliorate tissue regeneration and repair, thus retarding chronic fibrosis. In a study completed very recently, we found that MVs release by hWJ-MSCs improve aberrant kidney repair via HGF induction in damaged tubular cells. More evidence is needed to address this concern.
